# Origin of the human malaria parasite *Plasmodium falciparum* in gorillas

**DOI:** 10.1186/1475-2875-9-S2-I6

**Published:** 2010-10-20

**Authors:** Weimin Liu, Yingying Li, Gerald H Learn, Rebecca S Rudicell, Joel D Robertson, Brandon F Keele, Jean-Bosco N Ndjango, Crickette M Sanz, David B Morgan, Sabrina Locatelli, Mary K Gonder, Philip J Kranzusch, Peter D Walsh, Eric Delaporte, Eitel Mpoudi-Ngole, Alexander V Georgiev, Martin N Muller, George M Shaw, Martine Peeters, Paul M Sharp, Julian C Rayner, Beatrice H Hahn

**Affiliations:** 1 Department of Medicine, UnIversIty of Alabama at Birmingham, Birmingham, Alabama 35294, USA; 2 Department of Microbiology, University of Alabama at Birmingham, Birmingham, Alabama 35294, USA; 3 Department of Ecology and Management of Plant and Animal Resources, Faculty of Sciences, University of Kisangani, Democratic Republic of the Congo; 4 Department of Anthropology, Washington University, Saint Louis, Missouri 63130, USA; 5 Congo Program, Wildlife ConservationSociety,Brazzaville, B.P. 14537, Republic of Congo; 6 Lester E. Fisher Center for the Study and Conservation of Apes, Lincoln Park Zoo, Chicago, Illinois 60614, USA; 7 Department of Biological Sciences, University at Albany, State University of New York, Albany, New York 12222, USA; 8 Department of Microbiology and Molecular Genetics, Harvard Medical School, Boston, Massachusetts 02115, USA; 9 VaccinApe, Bethesda, Maryland, USA; 10 Institut de Recherche pour le Développement (IRD)and University of Montpellier 1,34394 Montpellier, France; 11 Institut de Recherches Médicales et d'études des Plantes Médicinales Prévention du Sida ou Cameroun, Centre de Recherche Médicale, BP 906, Yaoundé, Cameroun; 12 Department of Human Evolutionary Biology, Harvard University, Cambridge, Massachusetts, 02138, USA; 13 Department of Anthropology, University of New Mexico, Albuquerque, New Mexico, 87131, USA; 14 Institute of Evolutionary Biology, University of Edinburgh, Edinburgh EH9 3JT, UK; 15 Sanger Institute Malaria Programme, The Wellcome Trust Sanger Institute, Cambridge CB10 1SA, UK

## 

*Plasmodium falciparum* is the most prevalent and lethal of the malaria parasites infecting humans, yet the origin and evolutionary history of this important pathogen remain controversial. Here, we used single genome amplification (SGA) strategies to show that wild-living African apes are naturally infected with at least nine *Plasmodium* species, including one that is the direct precursor of *P.falciparum.* Among nearly 3,000 ape fecal specimens collected from 57 field sites throughout central Africa, we found *Plasmodium spp.* infection in chimpanzees (*Pan troglodytes*) and western gorillas (*Gorilla gorilla*)*,* but not in eastern gorillas (*Gorilla beringei*) or bonobos (*Panpaniscus*)*.* Ape plasmodial infections were highly prevalent, widely distributed, and almost always made up of mixed parasite species. To obtain *Plasmodium* sequences not confounded by *in vitro* recombination, we used SGA to amplify fragments of the mitochondrial (956bp of the *cytochrome b* gene; 3.4kb and 3.3kb half-genome fragments), apicoplast (390bp of the *caseinolytic protease C* gene) and nuclear (772bp of the *lactate dehydrogenase* gene) genomes. Among more than 1,100 such sequences from 80 chimpanzee and 55 gorilla samples, we found nine that were related to *P. malariae, P. ovale* or *P. vivax.* All others grouped within one of six chimpanzee- or gorilla-specific lineages representing distinct *Plasmodium* species within the *Laverania* subgenus. One of these from western gorillas was comprised of parasites that were nearly identical to *P. falciparum.* In phylogenetic trees of full-length mitochondrial sequences, human *P. falciparum* formed a monophyletic lineage within the gorilla parasite radiation. These findings indicate that *P. falciparum* is of gorilla origin and not of chimpanzee, bonobo or ancient human origin, and that all known human strains appear to have resulted from a single cross-species transmission event.

